# A Novel Recombinant Newcastle Disease Virus Vectored DIVA Vaccine against Peste des Petits Ruminants in Goats

**DOI:** 10.3390/vaccines8020205

**Published:** 2020-04-28

**Authors:** Magdalena Murr, Bernd Hoffmann, Christian Grund, Angela Römer-Oberdörfer, Thomas C. Mettenleiter

**Affiliations:** 1 Institute of Molecular Virology and Cell Biology, Friedrich-Loeffler-Institut, Federal Research Institute for Animal Health, Südufer 10, 17493 Greifswald-Insel Riems, Germany; 2 Institute of Diagnostic Virology, Friedrich-Loeffler-Institut, Federal Research Institute for Animal Health, Südufer 10, 17493 Greifswald-Insel Riems, Germany

**Keywords:** Newcastle disease virus, peste des petits ruminants, *small ruminant morbillivirus*, vector vaccine, DIVA, mammals

## Abstract

Peste des petits ruminants virus (PPRV, species: *small ruminant morbillivirus*) is the causative agent of the eponymous notifiable disease, the peste des petits ruminants (PPR) in wild and domestic sheep and goats. Mortality rates vary between 50% and 100%, causing significant losses of estimated 1.5 to 2 billion US Dollars per year. Live-attenuated PPRV vaccine strains are used in the field for disease prevention, but the application of a more thermostable vaccine enabling differentiation between infected and vaccinated animals (DIVA) would be highly desirable to achieve the goal of global disease eradication. We generated a recombinant Newcastle disease virus (rNDV) based on the live-attenuated NDV Clone 30 that expresses the surface protein hemagglutinin (H) of PPRV strain Kurdistan/11 (rNDV_H_Kur_). In vitro analyses confirmed transgene expression as well as virus replication in avian, caprine, and ovine cells. Two consecutive subcutaneous vaccinations of German domestic goats with rNDV_H_Kur_ prevented clinical signs and hematogenic dissemination after an intranasal challenge with virulent PPRV Kurdistan/11. Virus shedding by different routes was reduced to a similar extent as after vaccination with the live-attenuated PPRV strain Nigeria 75/1. Goats that were either not vaccinated or inoculated with parental rNDV were used as controls. In summary, we demonstrate in a proof-of-concept study that an NDV vectored vaccine can protect against PPR. Furthermore, it provides DIVA-applicability and a high thermal tolerance.

## 1. Introduction

Peste des petits ruminants virus (PPRV, species: *small ruminant morbillivirus*) belongs to the family Paramyxoviridae within the order Mononegavirales, which includes viruses with a single stranded, negative-sense, non-segmented RNA genome [1,2]. PPRV is closely related to rinderpest virus of cattle but represents a distinct virus species. The genome comprises about 15.9 kilobases (kb) and encodes viral proteins in the order 3′- nucleoprotein (N), phosphoprotein (P), matrix protein (M), fusion protein (F), hemagglutinin (H), RNA-dependent RNA-polymerase or large protein (L) -5’ [3,4,5]. It is the causative agent of peste des petits ruminants (PPR), a notifiable, highly contagious transboundary disease in wild and domestic small ruminants such as sheep and goats. PPR was first reported in 1942 in Ivory Coast, West Africa [6]. Since then, it has spread across northern and sub-Saharan Africa into Asia and has now been reported in over 70 countries in Africa, the Near and Middle East, and Central and Eastern Asia [7,8,9,10,11,12,13], including Georgia in 2016 [14] and Bulgaria in 2018 [15].

PPRV exists as a single serotype, but is classified into four genetically different lineages, according to F or N gene sequence identity. The geographic distribution of PPRV correlates with the genetic lineages. While lineage I–III viruses are mainly restricted to Africa, lineage IV viruses appear in increasing numbers in countries all over the endemic regions, although they were previously assigned exclusively to Asia [7,8,9,16,17]. Until the eradication of rinderpest (RP) in 2011, vaccination against RP was also performed in small ruminants, which were protected against RP, as well as against PPR, by cross-reactivity of induced antibodies. It is assumed that the cessation of vaccination as a consequence of its eradication contributed to the increasing number of PPR cases [18].

PPR exhibits high morbidity rates (90%–100%), and mortality rates between 50% and 100%, depending on the virus strain. Since 80% of the world’s sheep and goat populations are located in PPR-endemic regions, in which approx. 330 million households are depending on their livestock, PPR has a major impact on food supply and income, increasing malnutrition and poverty. Global financial losses due to PPR are estimated to be 1.5 to 2 billion US dollars per year. Since consumption of goat meat and milk products increases with a growing human population, the Food and Agriculture Organization (FAO) and the World Organization for Animal Health (OIE) set the goal of PPRV global eradication by 2030, following the rinderpest example [19]. 

Disease prevention occurs by vaccination with homologous live-attenuated PPRV strains, like Nigeria 75/1, which belongs to lineage II, and was attenuated by passaging in Vero cells [20,21]. For application in India, three vaccines, derived from lineage IV viruses, were licensed, namely Sungri/96, Arasur/87, and Coimbatore/97 [22]. However, these PPR vaccines are disadvantageous regarding their low thermal tolerance and inability to differentiate between vaccinated and infected animals, which is important regarding the sero-epidemiological surveillance following an eradication program and the determination of PPRV-free zones. This disadvantage can be overcome by the use of vaccines enabling differentiation between infected and vaccinated animals (DIVA), and their accompanying serological tests [23]. The first general DIVA vaccines were deletion mutants of wild type viruses. Serological tests aimed at the deleted gene or protein respectively, and were able to differentiate vaccinated from infected animals [24]. Viral vectored vaccines also apply to the DIVA-strategy as the vector virus expresses only one or two proteins of the respective wild type virus.

Recombinant vector viruses expressing the immunogenic surface proteins F or H of PPRV are able to provide protection against a PPRV infection by inducing PPRV-neutralizing antibodies. Vaccinated animals can by differentiated by the absence of PPRV N specific antibodies that are present in PPRV-infected animals. This can be achieved by the commercially available competitive PPR-N-ELISA [25]. Vaccination and challenge infection studies in sheep or goats report about the use of either capripoxviruses [26,27,28,29], adenoviruses [30,31,32,33,34], or vaccinia viruses [35] as vectors. However, none has been licensed for use so far.

We evaluated another vector virus, Newcastle disease virus (NDV), for its potential as a recombinant DIVA vaccine against PPR in goats. NDV (species: *Avian orthoavulavirus 1*) also belongs to the family Paramyxoviridae but is classified into the genus *Orthoavulavirus* [1,36]. Similar to PPRV, the single stranded, negative sense RNA genome encodes the viral proteins 3’- nucleoprotein (NP), phosphoprotein (P), matrix protein (M), fusion protein (F), hemagglutinin-neuraminidase (HN), and the RNA-dependent RNA-polymerase or large protein (L) -5’ [37]. The mRNAs for the non-structural proteins V and W emerge from the P gene during transcription by mRNA-editing [38,39]. Virulent NDV strains cause Newcastle disease (ND) in avian species. It was first described in 1926 in Java (Indonesia) and in 1927 in England [40], but it is meanwhile endemic in many countries in Africa, Asia, Central and South America, causing outbreaks in poultry with high mortality, and subsequently significant economic losses for poultry production. NDV strains are divided into three pathotypes, according to the severity of clinical signs they cause in chickens, which is assessed by the intracerebral pathogenicity index (ICPI) [41,42]. Commonly, lentogenic, non-virulent strains with an ICPI <0.7 are used as live-attenuated vaccines to control and prevent ND in poultry but also as a backbone for recombinant vector vaccines. The potential of NDV as a vector for the expression of heterologous proteins of other avian pathogens and as bivalent vaccine has been repeatedly shown [43,44,45,46,47,48,49,50]. Additionally, the safety and the immunogenicity of NDV in mammals was demonstrated for different species [51,52,53,54,55,56,57,58,59]. Due to its strong host range restriction, only minimal replication in non-host species is expected, resulting in the potential for a safe, attenuated vaccine. Furthermore, a pre-existing immunity, such as against capripoxviruses, cannot hamper vaccine efficacy, as avian and mammalian paramyxoviruses are genetically and serologically distinct.

The aim of the study was the development of a recombinant NDV (rNDV) vector vaccine that expresses the surface glycoprotein H of PPRV strain Kurdistan/11 (lineage IV). The protective efficacy of rNDV_H_Kur_ was investigated after a single or double immunization of goats and subsequent challenge infection with virulent PPRV Kurdistan/11 in comparison with the conventional live-attenuated PPRV vaccine Nigeria 75/1.

## 2. Materials and Methods 

### 2.1. Cells and Viruses

Chicken embryo fibroblasts (CEFs) were used for propagation and characterization of recombinant NDV. CEFs were prepared from 10-day-old specific pathogen free (SPF) embryonated chicken eggs (ECE), purchased from Valo, BioMedia (Osterholz-Scharmbeck, Germany) and incubated at 37 °C with 55% humidity. QM9 cells (quail muscle cells, clone 9, CCLV-RIE 466), SFT-R (sheep fetal thymus cells, CCLV-1976), and ZZ-R (fetal goat tongue mucosa cells, CCLV-1984) were used for virus characterization. CEF and QM9 cells were maintained and grown in minimal essential medium, supplemented with NaHCO_3_, Na-Pyruvate, non-essential amino acids, and 10% fetal calf serum (FCS). SFT-R cells were maintained and grown in minimal essential medium, supplemented with NaHCO_3_ and 10% FCS, whereas ZZ-R cells were maintained and grown in a mixture of Ham’s F12 and Iscove’s modified Dulbecco’s medium, supplemented with L-glutamine and 10% FCS. BSR-T7 (baby hamster kidney cells, BHK 21, clone BSR-T7/5; CCLV-RIE 582) [60], which stably express phage T7 polymerase, were used for recovery of recombinant NDV, and were maintained and grown in Glasgow minimal essential medium, supplemented with NaHCO_3_, casein peptone, meat peptone, yeast extract, essential amino acids, and 10% FCS.

Vero cells constitutively expressing the canine signaling lymphocyte activation molecule (SLAM)-receptor (VerodogSLAMtag, VDS) [61] were used for propagation of PPRV and virus neutralization assays. Cells were maintained and grown in minimal essential medium, supplemented with NaHCO_3_, Na-Pyruvate, non-essential amino acids, and 10% FCS in the presence of 1 mg of Zeocin/mL (Invitrogen, Carlsbad, CA, USA). All cells were incubated at 37 °C with 3%–5% CO_2_.

Recombinant NDV (rNDVGu) based on lentogenic NDV Clone 30 (Genbank Acc. No. Y18898) has been described before and is further on referred to as rNDV [62]. Live-attenuated PPRV vaccine strain Nigeria 75/1 and virulent PPRV challenge strain Kurdistan/11 were obtained from the National German Reference Laboratory for PPR (B. Hoffmann, Friedrich-Loeffler-Institut (FLI) Insel Riems).

### 2.2. Construction and Generation of Recombinant NDV Expressing PPRV Hemagglutinin (H)

**Figure 1:**
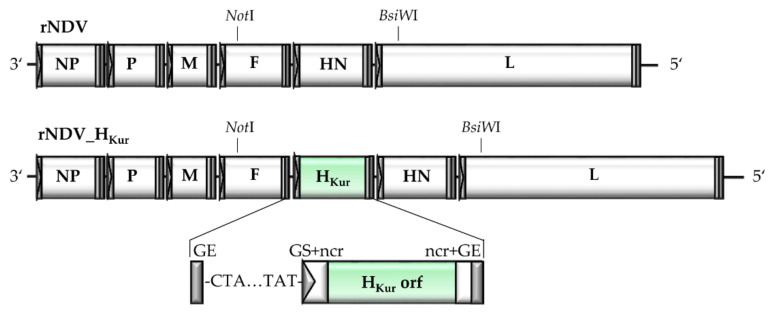
Construction of recombinant Newcastle disease virus (NDV) expressing peste des petits ruminants virus (PPRV) hemagglutinin (H). Schematic representation of genomic organization of parental rNDV and newly generated rNDV_H_Kur_ expressing PPRV hemagglutinin (H). The PPRV transgene is inserted between the genes encoding the NDV fusion protein (F) and the hemagglutinin-neuraminidase (HN). The open reading frame (orf) is flanked by the gene start sequence (GS, grey triangle) and the gene end sequence (GE, grey rectangle), and as indicated by the NDV HN-non-coding regions (ncr, white rectangles). The basis of virus rescue is the full-length plasmids pNDVGu und pNDV_H_Kur._ The 6035 bp *Not*I-*BsiW*I fragment of pUCNDV_H_Kur_ was inserted into pNDVGu after cleavage of the respective plasmids with *Not*I and *BsiW*I, resulting in pNDV_H_Kur._

Viral RNA was extracted from a virus stock of PPRV Kurdistan/11 using the Trizol LS procedure (Thermo Fisher Scientific, Carlsbad, CA, USA). The open reading frame (orf) encoding PPRV H was translated into cDNA and amplified using the Expand High Fidelity^PLUS^ PCR System (Roche Applied Science, Mannheim, Germany) with specific primers PPRHncrNDF (5’-CGCTTCACCGACAACAGTCCTCAATCCATGTCCGCACAAAGGGAAAGGATCAATGCC-3’) and PPRHncrNDR (5’-CATCTTTCCAACTCCTTAGTATAATTGACTTCAGACTGGATTACATGTTACCTCTATAC-3’). The 1.7 kb PCR product was gel-purified using the QIAquick^®^ Gel Extraction Kit (Qiagen, Hilden, Germany) and cloned into pGEM^®^-T Easy Vector (Promega, Madison, WI, USA), resulting in pGemPPRKurHncrND. A cloning vector (pUC-derivate) containing the 5903 bp *Not*I-*BsiW*I fragment of pNDVH5 [63] was altered by Phusion polymerase chain reaction (Finnzymes Phusion^®^, New England Biolabs^®^) [64] using megaprimer MePrGuPPRKurHncrND, generated by PCR with plasmid pGemPPRKurHncrND and primers PPRHncrNDF and PPRHncrNDR. The resulting plasmid pUCNDV_H_Kur_ contained the 6035 bp *Not*I-*BsiW*I fragment of pNDV_H_Kur_, which was inserted into pNDVGu after cleavage of both plasmids with *Not*I and *BsiW*I, resulting in the full-length plasmid pNDV_H_Kur_ (Figure 1).

### 2.3. Transfection and Virus Recovery

Infectious recombinant NDV was rescued and propagated as described [65]. The full-length plasmid pNDV_H_Kur_ was transfected together with helper plasmids pCiteNP, pCiteP, and pCiteL into BSR-T7 cells using Lipofectamine^®^3000 (Invitrogen) and a DNA to Lipofectamine ratio of 1.0 µg to 1.5 µL, following the manufacturer’s instruction. 

### 2.4. RNA Isolation, Reverse Transcriptase (RT)-PCR, and Sequencing

RNA was isolated from allantoic fluid after the first and second passage in ECE of recombinant NDV_H_Kur_ using the QIAamp^®^ Viral RNA Mini Kit (Qiagen). Genomic regions, encoding the proteolytic cleavage site within the NDV F gene, as well as the region, encoding the inserted PPRV H, were transcribed into cDNA and amplified, using the Transcriptor One-Step RT PCR Kit (Roche Applied Science, Mannheim, Germany). Sanger-sequencing (Sequencer 3130 Genetic Analyzer, Applied Biosystems, Foster City, CA, USA) was used to confirm virus identity. Virus stock was prepared from allantoic fluid of the second ECE passage.

### 2.5. Antibodies and Sera

Mouse monoclonal antibodies against NDV-HN (mAb NDV-HN) [66], a monospecific rabbit anti NDV-HN serum (α NDV-HN) [67], a monospecific rabbit anti NDV-F serum (α NDV-F) [62], a monospecific rabbit anti PPRV-H serum (α PPRV-H), as well as a hyperimmune serum against NDV (HIS α NDV), were used to detect viral proteins by indirect immunofluorescence assay (IFA) or Western blotting. Protein expression was visualized after binding of secondary Alexa Fluor^TM^ 488 α-rabbit or 568 α-mouse IgG goat antibody (Invitrogen), or peroxidase-conjugated species-specific secondary antibody (Dianova, Hamburg, Germany), respectively. 

### 2.6. Kinetics of Replication

CEF, QM9, SFT-R, and ZZ-R cells were infected with rNDV or rNDV_H_Kur_ at a multiplicity of infection (moi) of 0.01. Cell monolayers were washed twice with medium after an adsorption time of 40 min. Cell culture supernatants were harvested 0, 17, 24, 48, and 72 h after infection (p.i.), and examined for the presence of infectious progeny viruses by titration in duplicate on QM9, which were fixed 20 h p.i. Viral titers (50% tissue culture infectious dose, TCID_50_) were calculated by IFA using HIS α NDV and Alexa Fluor^TM^ 488 α-rabbit secondary antibody (Invitrogen) in two independent experiments. 

Differences between groups were analyzed using an unpaired *t*-test and a significance interval of 95% (α = 0.05). Graphs preparation and statistical analyses were performed using GraphPad Prism Software Version 7.05 (San Diego, CA, USA).

### 2.7. Western Blot Analyses

CEFs were infected with rNDV and rNDV_H_Kur_ at an moi of 5 and harvested 48 h p.i. Lysates of VDS cells infected with PPRV Kurdistan/11 at an moi of 1, which were harvested 3 and 4 days p.i., were provided by the German National Reference Laboratory for PPR. All cell lysates were denatured at 95 °C for 10 min, proteins were separated by sodium dodecyl sulphate polyacrylamide gel electrophoresis (SDS-PAGE), and subsequently transferred to nitrocellulose membranes. Viral proteins were detected by incubation with α PPRV-H, α NDV-HN, or α NDV-F. After primary antibody incubation, binding of peroxidase-conjugated species-specific secondary antibodies (Dianova) was detected by chemiluminescence substrate (Thermo Scientific, Rockford, IL, USA) and visualized by ChemiDoc XRS+ (BioRad, Hercules, CA, USA).

### 2.8. Indirect Immunofluorescence Assay

QM9 cells were infected with rNDV and rNDV_H_Kur_ at an moi of 0.1, fixed with 3.7% formaldehyde 24 h p.i., and permeabilized using 0.1% Triton X-100. After blocking of the cells with 5% bovine serum albumin (BSA) in PBS, they were incubated with α PPRV-H and monoclonal antibody (mAb) NDV-HN. Binding of primary antibody was visualized using Alexa 488 α-rabbit or 568 α-mouse secondary antibody (Invitrogen). 4′,6-Diamidino-2-phenylindole (DAPI) (Roth, Karlsruhe, Germany) was included in washing steps after binding of secondary antibodies to stain nuclei. Images were taken on a Leica SP5 confocal microscope (Leica Microsystems GmbH, Wetzlar, Germany) with an oil immersion objective (HCX PL APO 63×/1.40–0.60 objective). Sequential z-sections of stained cells were acquired for maximum projection, and images were processed using ImageJ software (Wayne Rasband, National Institutes of Health, USA).

### 2.9. Thermostability

Aliquots of rNDV_H_Kur_ containing allantoic fluid (500 µL each) were stored at different temperatures (−80 °C, −20 °C, 4 °C, 21 °C, 37 °C, 42 °C, and 45 °C) for two weeks. Aliquots were removed after 0, 1, 2, 3, 4, 7, 8, 9, 10, and 14 days of incubation for determining the hemagglutination (HA) activity according to the standard protocol [63], as well as after 0, 1, 2, 7, and 14 days for determining the infectious virus titer (TCID_50_) by titrating on QM9 cells.

### 2.10. Animal Trials

The determination of the intracerebral pathogenicity index (ICPI) in one-day-old SPF-chickens as well as a vaccination experiment followed by a challenge infection using 5-month-old goats of a German domestic breed were carried out in biosafety level (BSL)-3 animal facilities at the Friedrich-Loeffler-Institut, Insel Riems. All animal experiments were approved by the animal welfare committee (Landesamt für Landwirtschaft und Fischerei Mecklenburg-Vorpommern, Thierfelder Straße 18, 18059 Rostock, LALLF MV/TSD/7221.3-1-005/19 and LALLF M-V/TSD/7221.3-1-008/19) and supervised by the commissioner for animal welfare at the FLI, representing the institutional Animal Care and Use Committee (ACUC).

#### 2.10.1. Determination of Pathogenicity of rNDV_H_Kur_ in vivo

The ICPI was determined in one-day-old SPF chickens, following the standard protocol [68]. Ten one-day-old SPF chickens were inoculated intracerebrally with 100 µL of a 10^-1^ dilution of rNDV_H_Kur_. Mortality and clinical signs were monitored daily for 8 days. Chickens showing severe clinical distress during the experiment were euthanized immediately. Criteria for euthanasia were either dyspnea, apathy, somnolence, akinesia, or deficit motor activity, respectively.

#### 2.10.2. Vaccination and Challenge Infection Experiment

In total, 25 goats were divided into five groups of five animals each. The first group (no. 25–29) remained unvaccinated and served as a negative control, while goats of the second group (no. 20–24) were inoculated twice with 2 × 10^6.5^ TCID_50_/mL of rNDV in an interval of three weeks as an empty vector control. Another five goats (group 3, no. 15–19) were vaccinated with 10^3.83^ TCID_50_/mL of live-attenuated PPRV vaccine strain Nigeria 75/1 as positive vaccine control. The animals of the test groups received either one (group 4, no. 10–14) or two vaccinations (group 5, no. 05–09) in an interval of three weeks with 2 × 10^6.5^ TCID_50_/mL of rNDV_H_Kur_ (Table 1). All vaccines were inoculated subcutaneously (s.c.) into the neck. Blood and nasal swabs were taken from vaccinated animals 1 and 2 days post vaccination (dpv) to monitor vaccine virus excretion. Serum was taken on the same days, and on 28 and 35 dpv from goats vaccinated once with rNDV_H_Kur_ (group 4). Furthermore, body temperatures were recorded daily. Animal no. 15 of group 3 was euthanized on day 30 because it was febrile and apathetic. Animal no. 09 of group 5 died spontaneously on day 34 of the trial. Vaccination was excluded as the cause for these cases of death because fever was recorded already before vaccination of the respective animals, and also, other non-vaccinated goats were affected but recovered.

All goats were intranasally inoculated with virulent PPRV strain Kurdistan/11 (Table 1) using nasal atomizers (LMA MAD^TM^ 100, Wolfram Droh GmbH, Mainz, Germany) three weeks after the prime immunization (groups 3 and 4) or the prime-boost immunization (groups 2 and 5), respectively. Clinical signs, including body temperature, were monitored and recorded daily for three weeks after the challenge infection. Severity of clinical signs was evaluated from 0 to 3 in the categories: general health condition, body temperature, oculo-nasal discharge, mucosal oral lesions, defecation, and respiratory signs. A score of 0 was annotated to “no clinical signs”, a score 1 to “mild clinical signs”, a score 2 to “high temperature and moderate clinical signs”, and a score 3 to “severe clinical signs” as described before [69,70]. The individual daily clinical score (CS) of each animal represents the cumulative scores of all categories. Animals with severe clinical distress or reaching the humane endpoint during the experiment were euthanized immediately. Criteria for euthanasia were defined as a CS of 20 in total, or a CS of 4 in “general health condition”, a CS of 3 at two consecutive days in “general health conditions“ and a CS ≥10 in other categories, or a CS of 2 in “general health conditions” at two consecutive days and a CS ≥15 in other categories. 

Blood, nasal swabs, ocular swabs, oral swabs, and fecal swabs were taken from all animals 3, 5, 7, 9, 11, 14, 17, 21, and 28 days post challenge (dpc) to monitor viremia, shedding, and replication of the challenge virus. Sera for analyses of PPRV and NDV specific antibodies were taken on the same days. 

All five goats of the empty vector control group (group 2) and three of five goats of the negative control group (group 1) were euthanized 9 dpc; the remaining two goats of group 1 were euthanized 14 dpc, as they reached the humane endpoint. All remaining goats were euthanized 28 dpc, determining the end of the trial. Organ samples from the lung, liver, spleen, and mediastinal and mesenterial lymph nodes from all animals were taken postmortem for analysis of the presence of PPRV genome.

### 2.11. Virus Shedding

Viral RNA from blood and swabs was isolated automatically (KingFisher 96 Flex, ThermoFisher, Waltham, MA, USA) using the NucleoMag^®^ VET Kit (Machery-Nagel, Düren, Germany). Reverse transcriptase quantitative real-time PCR (RT-qPCR) was used to detect RNA, specific to NDV NP [62] or PPRV N [71] after vaccination and challenge infection, respectively. Detected virus loads were indicated as genomic equivalents (GEQ) using calibration curves of defined RNA standards, which were included in each RT-qPCR run. All RT-qPCR assays were combined with a heterologous internal control system, described for the detection of pestiviruses [72]. All RT-qPCRs were performed in 12.5 μL volumes using the AgPath-ID RT-PCR Kit (Ambion, Austin, TX, USA) and run on a Mx3005P thermocycler machine (Agilent, Santa Clara, CA, USA). Cycle threshold (Ct) values of 40 were set as the cut-off, representing a GEQ/mL of 50 (PPRV N RT-qPCR) or 1000 (NDV NP RT-qPCR).

Differences between groups were analyzed using the Kruskal–Wallis test for comparison of unmatched groups and a significance interval of 95% (α = 0.05). Correction for multiple comparison was performed using Dunn’s test. The *p-*values are indicated as <0.05. Graphs and statistical analyses were performed using GraphPad Prism Software Version 7.05 (San Diego, CA, USA).

### 2.12. Serology

Serum antibody titers against NDV NP (ID Screen^®^ Newcastle Disease Competition) and PPRV N (ID Screen^®^ PPR Competition) were determined using commercial competitive ELISA (ID.Vet, Grabels, France).

To detect PPRV-neutralizing serum antibodies in goats, vaccinated with rNDV_H_Kur_ or PPRV Nigeria 75/1, after vaccination and after PPRV challenge infection, a virus neutralization test (VNT) was performed according to the standard protocol [73]. Sera were tested in duplicates for neutralization activity against PPRV strain Nigeria 75/1. Sera were not titrated further than eight dilution steps, representing a maximal titer of 640.

## 3. Results

### 3.1. Generation of Recombinant NDV Expressing PPRV H

Recombinant NDV_H_Kur_ harbors a transgene encoding the H orf of virulent PPRV strain Kurdistan, isolated in 2011 from clinically ill wild goats in the Kurdish region of Iraq. It was assigned to lineage IV [70,74]. The foreign gene was inserted into the intergenic region of F and HN genes of lentogenic NDV strain Clone 30 (rNDV) with appropriate gene start, gene end and non-coding sequences derived from the NDV HN gene (Figure 1).

Recombinant NDV_H_Kur_ was recovered after transfection of the respective full-length plasmid together with helper plasmids pCite NP, pCite P, and pCite L into BSR T7/5 cells. Virus propagation was performed in SPF-ECE. After two passages in SPF-ECE, the identity of recombinant NDV_H_Kur_ was confirmed by amplification and sequencing of selected regions of the viral genome. Furthermore, rNDV_H_Kur_ was passaged ten times in CEFs. RNA of the 5th and 10th passage was isolated, and the genomic regions encoding the proteolytic cleavage site of NDV F, as well as PPRV H, were sequenced. No alteration was observed in any of the analyzed sequences (data not shown). These results suggest stability of the PPRV H insert over ten CEF passages.

### 3.2. In vitro Analyses of PPRV H Expression

**Figure 2:**
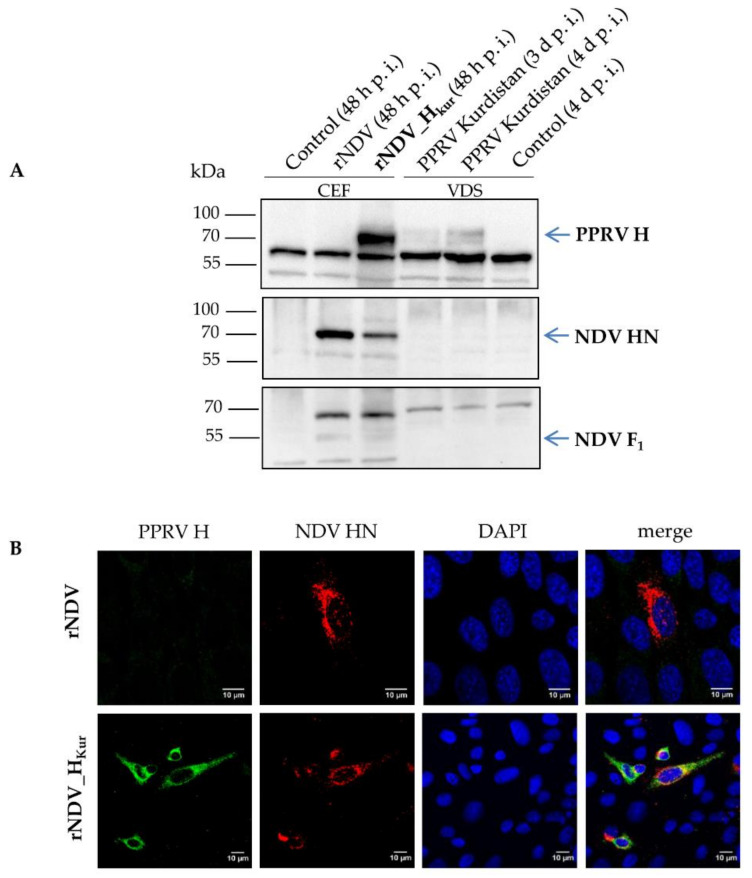
In vitro analyses of PPRV H expression. (**A**) Chicken embryo fibroblasts (CEFs) were infected with rNDV or rNDV_H_Kur_ (multiplicity of infection (moi) 5) and harvested 48 h post infection (p.i.). Vero dog SLAM cells (VDS) were infected with PPRV Kurdistan/11 (moi 1) and harvested 3 or 4 days p.i. The cell lysates were subjected to SDS-PAGE and subsequently to Western blot analysis. Viral proteins were visualized by immunostaining with rabbit antisera against PPRV-H, NDV-HN, and NDV-F. Binding of primary antibodies was detected after incubation with peroxidase-conjugated species-specific secondary antibodies by chemiluminescence substrate (Thermo Scientific). Identified proteins are indicated on the right. The molecular weights of marker proteins (PAGE Ruler™ Prestained Protein Ladder (Thermo Scientific)) are indicated on the left. (**B**) Quail muscle cells (QM9) were infected with rNDV or rNDV_H_Kur_ (moi 0.1), fixed 24 h p.i. and immunostained with monoclonal mouse antibodies against NDV-HN (red) and a rabbit antiserum against PPRV-H (green). Nuclei appear blue by 4′,6-diamidino-2-phenylindole (DAPI) staining. After Alexa 568 α-mouse and 488 α-rabbit secondary antibody (Invitrogen) incubation, viral protein expression and cellular distribution were analyzed by confocal microscopy.

Expression of PPRV H was investigated by Western blot analyses of CEFs, infected with parental rNDV and newly generated rNDV_H_Kur_. The α PPRV-H serum detected PPRV H with a molecular mass of about 70 kDa but did not react with NDV proteins or uninfected cells (Figure 2A). VDS cells infected with PPRV Kurdistan/11 verified the specificity of the signal. A signal of about 65 kDa was detected in all samples, which was classified as an unspecific reaction of the antiserum with cellular components. Expression of NDV subunit F_1_ (~55 kDa) and HN (~70 kDa) could be confirmed in NDV-infected CEFs but, as expected, not in PPRV-infected VDS cells (Figure 2A).

Furthermore, rNDV_H_Kur_-infected QM9 cells displayed specific staining of NDV HN and PPRV H in the cytoplasm and on the surface of infected cells, as shown by confocal microscopy (Figure 2B). Additionally, both proteins colocalized in the perinuclear area, presumably at the rough endoplasmic reticulum. In contrast, rNDV-infected cells were only positive for NDV HN and lacked reactivity with the PPRV H antiserum.

### 3.3. In vitro Replication in Cell Lines Originated from Different Species 

**Figure 3:**
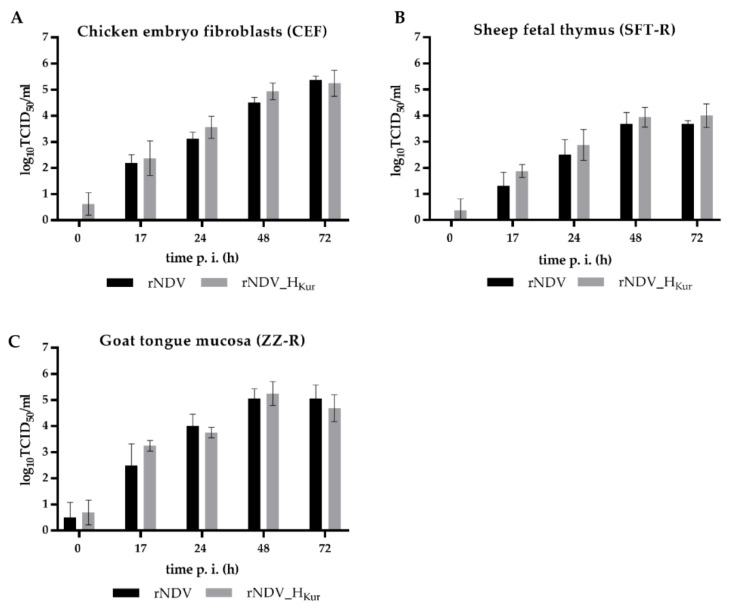
In vitro replication in cell lines originated from different species. (**A**) Chicken embryo fibroblasts (CEFs), (**B**) ovine fetal thymus cells (SFT-R), or (**C**) caprine tongue mucosa cells (ZZ-R) were infected with rNDV or rNDV_H_Kur_ (moi 0.01). Cell culture supernatants were harvested at indicated time points after infection (p.i.) and titrated on quail muscle (QM9) cells. Viral titers (TCID_50_/mL) were determined by subsequent immunostaining using a hyperimmune serum against NDV and Alexa 488 α-rabbit secondary antibody (Invitrogen). Bar charts depict mean viral titers ± standard deviation (*n* = 4, two samples each from two independent experiments).

Replication of rNDV_H_Kur_ and parental rNDV was investigated in avian cell lines of NDV host species (CEF, QM9) and in two mammalian cell lines of PPRV host species (SFT-R and ZZ-R). Both viruses were able to replicate in chicken fibroblasts (Figure 3A), but also in ovine thymus cells (Figure 3B) and caprine mucosal tongue cells (Figure 3C) with very similar titers. Final virus titers were about 10^5^ TCID_50_/mL in CEF and ZZ-R cells and about 1 log_10_ lower in SFT-R cells 72 h p.i.

No replication was observed in QM9 cells typical for lentogenic NDV, which are dependent on trypsin activation of F for in vitro replication (data not shown).

### 3.4. Investigation of Thermostability of rNDV_H_Kur_

Live-attenuated PPRV vaccine strains are rather thermosensitive [75], and therefore a continuous cold chain during transport has to be realized. Additional lyophilization of the vaccine seed leads to a loss in infectious viral titers. Therefore, aliquots of rNDV_H_Kur_ stock were incubated at different temperatures (−80, −20, 4, 21, 37, 42, and 45 °C) for 14 days. Virus integrity and infectivity was measured at indicated time points by hemagglutination (HA) test and by virus titration.

**Figure 4:**
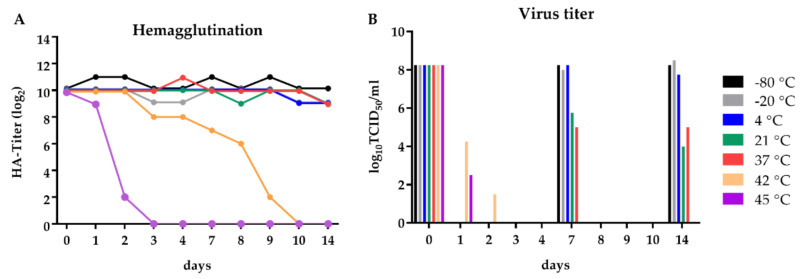
Investigation of thermostability of rNDV_H_Kur_. Aliquots of allantoic fluid containing rNDV_H_Kur_ (500 µL each) were stored at different temperatures (−80 °C, −20 °C, 4 °C, 21 °C, 37 °C, 42 °C, and 45 °C) for 14 days. At indicated time points, (**A**) the hemagglutination (HA) titer and (**B**) the infectious viral titers (TCID_50_/mL) were determined. The charts depict the results of a single experiment; the HA titer were determined in duplicates.

The HA titer remained stable at about 2^10^ for the observation period of 14 days at temperatures between −80 °C and 37 °C (Figure 4A). At 42 °C, the HA titers remained high between 2^10^ and 2^8^ for four days and dropped to 0 after ten days of incubation, whereas no HA activity was detectable at 45 °C already after three days. 

The infectious titer of rNDV_H_Kur_ (10^8.25^ TCID_50_/mL) remained stable at −80 °C, −20 °C, and 4 °C during the observation period (Figure 4B). At 21 °C and 37 °C, the TCID_50_/mL dropped to about 10^5^ after one week and remained at the same level for another week. The same virus activity was reached at 42 °C after only one week. Virus activity of 10^2.75^ TCID_50_/mL remained at 45 °C after one day of incubation.

### 3.5. Determination of Pathogenicity In Vivo 

An important prerequisite for a live NDV vaccine is its lentogenic pathotype for poultry, which is assessed by the ICPI in one-day-old SPF chickens. Recombinant NDV_H_Kur_ yielded a value of 0.088, indicating that the insertion of the foreign transgene did not increase pathogenicity of the recombinant virus.

### 3.6. Vaccination of Goats 

In order to evaluate the vaccine potential of rNDV_H_Kur_, five goats each were vaccinated either once (group 4) or twice (group 5) with 2 × 10^6.5^ TCID_50_/animal of rNDV_H_Kur_. To verify the results, three control groups were included. A non-vaccinated control group (group 1), an empty vector control group, which received 2 × 10^6.5^ TCID_50_/animal of parental rNDV (group 2), and a positive vaccine control group, which received the conventional live-attenuated PPRV vaccine strain Nigeria 75/1 (group 3) (Table 1). All inocula were administered s.c. into the neck. The site of inoculation remained inconspicuous after each vaccination, and none of the vaccinated goats showed any clinical signs. None of the animals, vaccinated with rNDV_H_Kur_, exhibited NDV specific RNA in the blood or the nasal swabs on the days following each vaccination, indicating limited spread of the NDV vectored vaccine virus in the animals. 

Some animals occasionally exhibited fever and digestive problems during the first days of the trial, which were considered not related to the vaccination, but rather to the general health conditions of the goats, as non-vaccinated animals were also affected. They were provided with licking stones supplemented with minerals and straw next to their general feed pellets to support digestion. Nevertheless, animal no. 15 of group 3 and animal no. 09 of group 5 were excluded from the trial for ethical reasons. The remaining goats recovered, and body temperatures remained below 39.5 °C until the challenge infection. 

**Figure 5:**
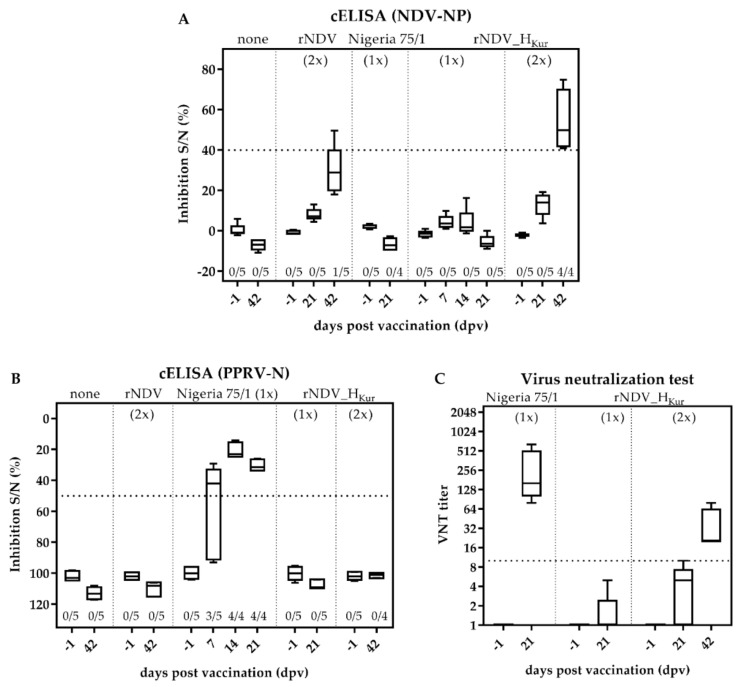
Serology after vaccination. Five goats each were either left unvaccinated (group 1), double inoculated with rNDV (group 2), single vaccinated with PPRV Nigeria 75/1 (group 3), single vaccinated with rNDV_H_Kur_ (group 4), or double vaccinated with rNDV_H_Kur_ (group 5). Sera were taken at indicated days after vaccination (dpv) and analyzed for the presence of antibodies, specific to either (**A**) NDV NP or (**B**) PPRV N by competitive ELISA (cELISA). A dotted line indicates the respective thresholds. The number of seropositive tested samples and the number of total tested samples are given below the boxplots. (**C**) Sera from goats, vaccinated with PPRV Nigeria 75/1 and rNDV_H_Kur_, were tested for the presence of neutralizing antibodies against PPRV Nigeria 75/1 by virus neutralization test (VNT). Sera exhibiting a VNT-titer >10 are considered seropositive (dotted line).

Sera from all goats were tested for antibodies against NDV NP and PPRV N by competitive ELISA (Figure 5A,B). As expected, none of the goats exhibited antibodies against NDV or PPRV prior to vaccination. Interestingly, only one animal of group 2 seroconverted after two inoculations with rNDV, whereas all goats, which were prime-boost vaccinated with rNDV_H_Kur_ (group 5), exhibited an NDV specific immunity 42 dpv. A single vaccination with recombinant rNDV_H_Kur_ did not elicit a detectable immune response (group 4) (Figure 5A). In contrast, all animals which were single vaccinated with PPRV Nigeria 75/1 exhibited PPRV N specific antibodies 14 dpv, whereas all other goats remained seronegative (Figure 5B). In order to determine whether vaccination with rNDV_H_Kur_ induced PPRV H specific antibodies, sera from goats vaccinated with rNDV_H_Kur_ once (group 4) or twice (group 5) were tested for their ability to neutralize PPRV Nigeria 75/1, compared to goats vaccinated with PPRV Nigeria 75/1 (group 3) (Figure 5C). Neutralizing antibodies against PPRV H with titers ranging between 20 and 80 were detectable three weeks after a second inoculation with rNDV_H_Kur_, whereas titers did not exceed the threshold of 10 after a single inoculation. Vaccination with PPRV Nigeria 75/1 elicited neutralizing antibody titers between 80 and 640 three weeks after immunization. 

### 3.7. Challenge Infection of Goats with Virulent PPRV Kurdistan/11

The intranasal challenge infection with virulent PPRV strain Kurdistan/11 was performed three weeks after the last immunization (Table 1).

**Figure 6:**
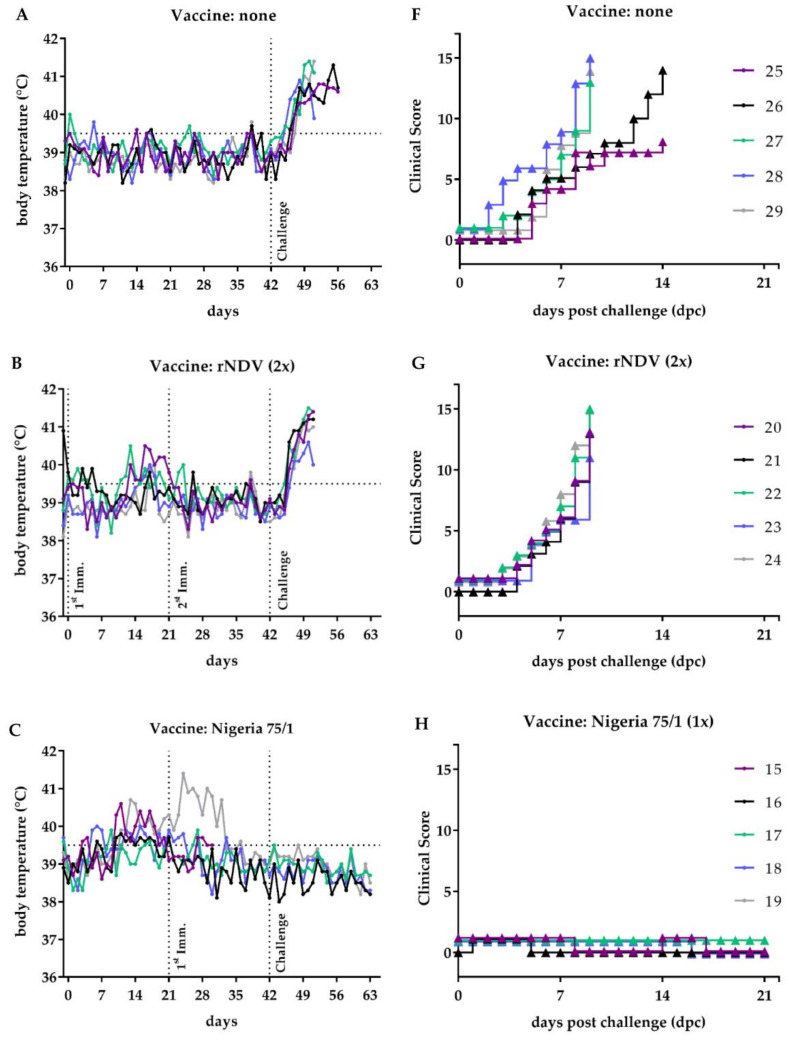

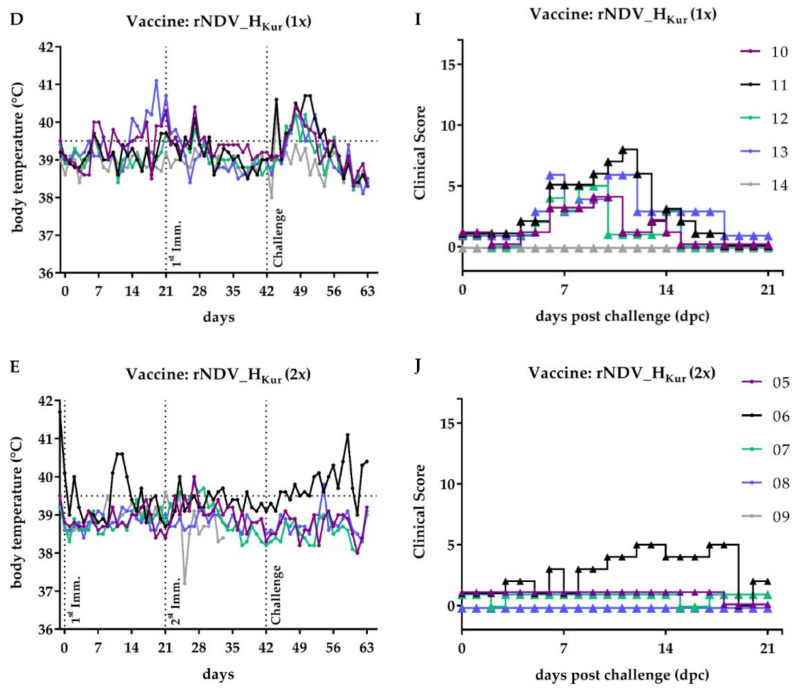
Body temperature and clinical course. (**A,F**) Five goats each were either left unvaccinated, (**B,G**) double inoculated with rNDV, (**C,H**) single vaccinated with PPRV Nigeria 75/1, (**D,I**) single vaccinated with rNDV_H_Kur_, or (**E,J**) double vaccinated with rNDV_H_Kur_. All goats were infected with PPRV Kurdistan/11 three weeks after immunization. (**A–E**) Rectal body temperatures were monitored daily, beginning one day prior to the first immunization. (**F–J**) Clinical signs were assessed daily for three weeks after challenge infection and the clinical scores calculated as described in Section 2.

All non-vaccinated and all rNDV-vaccinated goats of group 1 and 2 developed pronounced pyrexia, starting with elevated body temperatures 3 dpc, which increased up to 41 °C 9 dpc (Figure 6A,B). Additionally, goats exhibited specific PPR clinical signs as mucopurulent ocular and nasal discharge and ulcerations in the oral mucosa. Overall, the animals were lethargic and depressive, and partly too weak to stand. Typical clinical signs associated with PPR, such as diarrhea or respiratory disorders, were observed in some animals but were not dominant. Eight of the ten animals from these two groups reached the humane endpoint 9 dpc with CS ranging between 11 and 15 and were euthanized. The remaining two animals of group 1 (no. 25,26) were euthanized 14 dpc reaching the criteria for euthanasia (Figure 6F,G).

In contrast, all goats immunized with PPRV Nigeria 75/1 were protected from virulent PPRV Kurdistan/11 infection. None of the goats developed fever or a CS higher than 1, which was caused by temporary watery ocular discharge (Figure 6C,H). Noticeably, the same clinical picture was observed for animals vaccinated twice with rNDV_H_Kur_ (group 5) (Figure 6E,J). Three of them exhibited watery ocular discharge, and one animal did not show any clinical signs, resulting in very low CS in this group. The only exception was goat no. 06, which exhibited elevated body temperatures and slight depression. However, this animal developed fever from the beginning of the trial and was otherwise attentive. The shedding data described below revealed that clinical signs were not related to PPRV infection.

Four out of five goats of group 4, vaccinated once with rNDV_H_Kur_, developed PPR specific but strongly reduced clinical signs, compared to the negative control groups. The fifth goat in this group showed no clinical signs. Sick goats developed a fever over a period of nine days starting 4 dpc (Figure 6D), accompanied by watery ocular discharge and watery feces, observed for two of them. The CS ranged between 4 to 8 at its peak that was observed between 9 and 11 dpc (Figure 6I). Subsequently, they all recovered and were inconspicuous at the end of the trial.

### 3.8. Virus Dissemination and Shedding after PPRV Challenge Infection 

**Figure 7:**
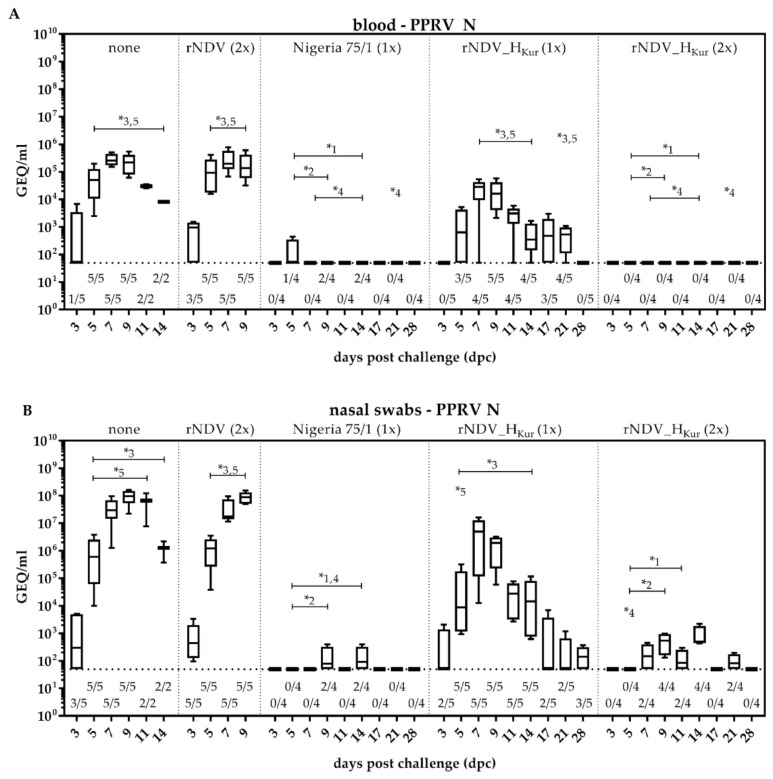

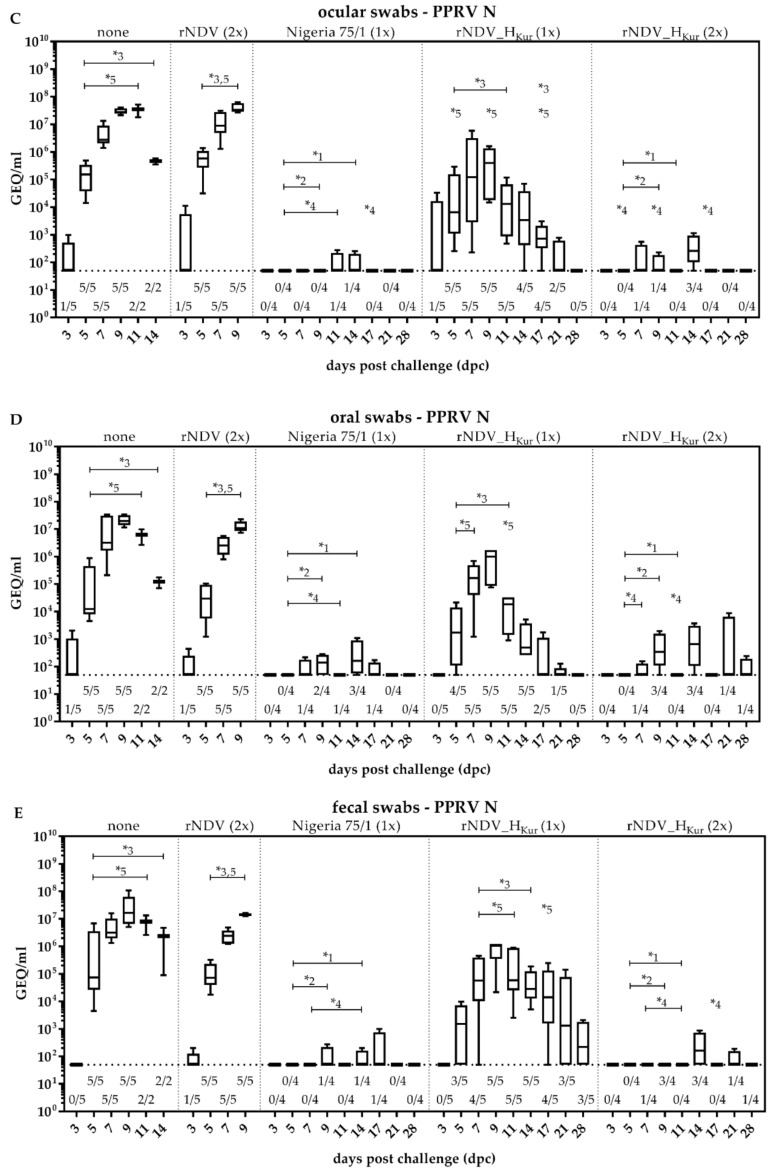
Virus dissemination and shedding after PPRV challenge infection. Five goats each were either left unvaccinated (group 1), double inoculated with rNDV (group 2), single vaccinated with PPRV Nigeria 75/1 (group 3), single vaccinated with rNDV_H_Kur_ (group 4), or double vaccinated with rNDV_H_Kur_ (group 5). All goats were infected with PPRV Kurdistan/11 three weeks after immunization. (**A**) Blood, (**B**) nasal swabs, (**C**) ocular swabs, (**D**) oral swabs, and (**E**) fecal swabs were taken at indicated days after challenge infection (dpc) and analyzed by quantitative real-time RT-PCR (RT-qPCR) for the presence of PPRV N specific RNA. Values were transformed to genome equivalents (GEQ) using calibration curves of defined RNA standards that were included within each RT-qPCR run. The number of positive samples relating to the total number of samples tested by RT-qPCR is given below the boxplot. Asterisks (*) and numbers above datasets indicate significant differences (α = 0.05) between respective groups at the same day.

Virulent PPRV challenge infection induced viremia in all negative control goats, starting 3 dpc with one positive animal in group 1 and three positive goats in group 2 (Figure 7A). The viral loads in the blood of the goats, euthanized 9 dpc, remained high up to 10^5^ GEQ/mL, while a decline in the two remaining control goats, no. 25 and 26, was observed 11 and 14 dpc. 

All goats of group 1 and 2 shed high amounts of challenge virus via the ocular, nasal, oral, and fecal route, displaying GEQ titers between 10^6^ and 10^8^/mL (Figure 7B–E). The quantities did not differ between non-vaccinated and rNDV-vaccinated control goats. The strongest onset of shedding 3 dpc was observed in the nasal swabs, the site of challenge virus inoculation with eight out of ten positive animals, while only two ocular and oral swabs, and one fecal swab were positive on the same day. Parallel to the decline in viral loads observed in the blood of goats no. 25 and 26 (control group 1), decreasing quantities of challenge virus were detected in all swab samples 14 dpc of the named animals.

In contrast, viremia was not observed in the animals, which were vaccinated twice with rNDV_H_Kur_ (group 5) and in goats, vaccinated with PPRV Nigeria 75/1 (group 3), except for one animal whose blood sample was positive on 5 dpc (Figure 7A).

In line with these results, all goats from both groups displayed significantly less viral RNA in all tested swabs between 5 dpc and 14 dpc (group 3) or 11 dpc (group 5), respectively, compared to non-vaccinated control goats (group 1) (Figure 7B–E). Both types of vaccines could not prevent shedding of challenge virus completely, as GEQ titers of 10^2^/mL in ocular, nasal, and fecal swabs and 10^3^ GEQ/mL in oral swabs were detected for some animals in both groups. However, a significant difference in viral loads between these two groups was not recorded.

Single rNDV_H_Kur_-immunized goats (group 4) developed viremia, starting 5 dpc to 21 dpc with significantly higher GEQ titers (10^2^–10^4^/mL) between 7 dpc and 14 dpc and on 21 dpc, compared to goats of group 3 and 5. No significant difference was detected compared to viral loads in the blood of non-vaccinated or empty vector control goats (group 1 and 2) (Figure 7A).

Significantly higher amounts of challenge virus were detected in swabs, taken from rNDV_H_Kur_-vaccinated goats after single vaccination (group 4) compared to those from animals of group 3 and 5. The determined GEQ titers ranged up to 10^6^/mL and peaked in the nasal swabs on 7 dpc and in the oral swabs, as well as ocular and fecal swabs on 9 dpc. None of these animals were positive anymore in ocular and oral swabs 28 dpc, and the amounts in nasal and fecal swabs declined to 10^2^–10^3^ GEQ/mL (Figure 7B–E).

### 3.9. Serology after PPRV Challenge Infection

**Figure 8:**
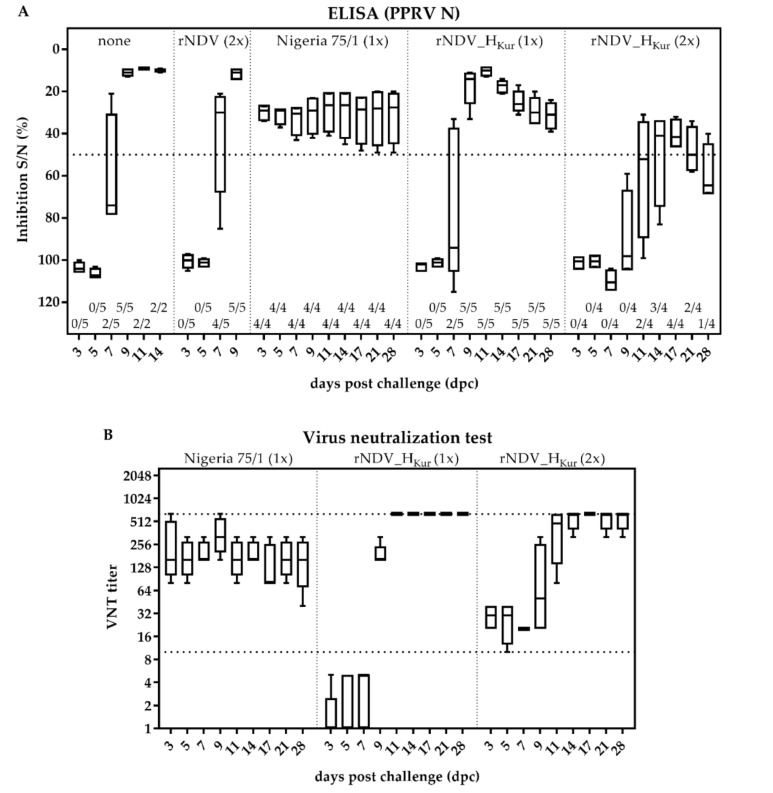
Serology after PPRV challenge infection. Five goats each were either left unvaccinated (group 1), double inoculated with rNDV (group 2), single vaccinated with PPRV Nigeria 75/1 (group 3), single vaccinated with rNDV_H_Kur_ (group 4), or double vaccinated with rNDV_H_Kur_ (group 5). All goats were infected with PPRV Kurdistan/11 three weeks after immunization. (**A**) Sera were taken at indicated days after challenge infection (dpc) and analyzed for the presence of antibodies, specific to PPRV N, by competitive ELISA (cELISA). A dotted line indicates the respective thresholds. The number of seropositive tested samples, relating to the total number of tested samples, is given below the boxplots. (**B**) Sera from goats, vaccinated with Nigeria 75/1 or rNDV_H_Kur_, were tested for the presence of neutralizing antibodies against PPRV Nigeria 75/1 by virus neutralization test (VNT). Sera exhibiting a VNT-titer >10 are considered seropositive (dotted line), sera can reach a maximum titer of 640, as they were not further titrated (dotted line).

Sera from all goats were retested for the presence of PPRV N specific antibodies at several dpc (Figure 8A). All goats of group 1 and 2 seroconverted within 9 dpc, whereas the animals of group 3, which had been immunized with PPRV Nigeria 75/1, exhibited PPRV N specific antibodies already before challenge infection. The degree of inhibition measured by the competitive ELISA stayed at the same level until 28 dpc, demonstrating that the conventional PPR vaccine is not applicable as a DIVA vaccine since vaccinated and infected animals are serologically not distinguishable. In contrast, none of the goats vaccinated with rNDV_H_Kur_ exhibited PPRV N specific antibodies before challenge infection. Seroconversion of single-vaccinated goats of group 4 was observed on 9 dpc and of group 5 on 17 dpc, characterized by a lower antibody level. Furthermore, it was noticed that level of antibodies started to decrease in single rNDV_H_Kur_-vaccinated goats (group 4) on 14 dpc and in double rNDV_H_Kur_-vaccinated goats (group 5) on 21 dpc. While all animals of group 4 remained seropositive, seroprevalence of group 5 declined to 25% on 28 dpc.

Titers of neutralizing antibodies, representing PPRV H specific antibodies in goats vaccinated with rNDV_H_Kur_ (group 4 and 5), increased rapidly after challenge infection, and in contrast to the PPRV N antibodies, stayed high at a maximal titer of 640 until 28 dpc (Figure 8B). Neutralizing antibody titers, obtained in sera from goats, vaccinated with PPRV Nigeria 75/1 (group 3), were not boosted further after challenge infection and remained constant, compared to the titers observed after vaccination, ranging between 80 and 640 with a median of 160.

### 3.10. Analyses of Postmortem Organ Samples 

**Figure 9:**
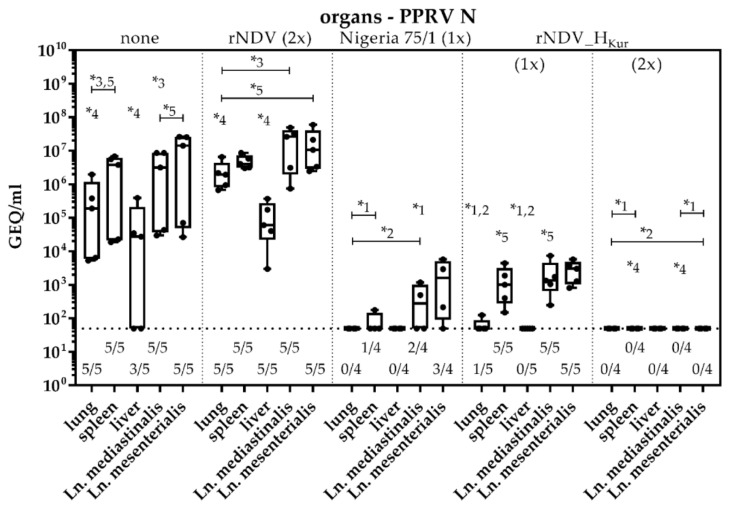
Analyses of postmortem organ samples. Five goats each were either left unvaccinated (group 1), double inoculated with rNDV (group 2), single vaccinated with PPRV Nigeria 75/1 (group 3), single vaccinated with rNDV_H_Kur_ (group 4), or double vaccinated with rNDV_H_Kur_ (group 5). All goats were infected with PPRV Kurdistan/11 three weeks after immunization. Goats from group 1 and 2 were euthanized on day 9 post challenge infection (dpc), except for two animals from group 1, which were euthanized 14 dpc. All goats from group 3, 4 and 5 were euthanized 28 dpc. Samples from lung, spleen, liver, and mediastinal and mesenterial lymph nodes were analyzed by quantitative real-time RT-PCR (RT-qPCR) for the presence of PPRV N specific RNA. Values were transformed to genome equivalents (GEQ) using calibration curves of defined RNA standards that were included within each RT-qPCR run. Boxplots also depict individual values for a better overview. The number of positive samples relating to the total number of samples tested by RT-qPCR is given below the boxplot. Asterisks (*) and numbers above datasets indicate significant differences (α = 0.05) between respective groups at the same day.

Organ samples from lung, liver, spleen, and mediastinal and mesenterial lymph nodes from all goats were analyzed postmortem for the presence of PPRV N specific RNA (Figure 9).

All investigated organ samples from goats of groups 1 and 2 tested positive, exhibiting lower GEQ titers in the liver (10^3^–10^5^/mL) and higher titers in the other organs (10^5^–10^7^ GEQ/mL), with the exception of goats no. 25 and 26 which lived until 14 dpc and whose livers were already cleared from PPRV. Accordingly, quantities of PPRV RNA in the other organs of these two animals ranged between 10^3^–10^4^ GEQ/mL and were lower, compared to the remaining control animals. The spleen of one animal, vaccinated with Nigeria 75/1 (group 3), tested positive for PPRV RNA, as well as the mediastinal and mesenterial lymph nodes of two or three goats, respectively. Viral loads ranged between 10^2^–10^3^ GEQ/mL. Since the RT-qPCR is not able to differentiate the PPRV vaccine from the challenge strain, and hematogenic dissemination and shedding of PPRV was almost absent after challenge infection in these animals, it is most likely remaining RNA of the PPRV vaccine virus that is still detectable in the lymphatic tissue. This is supported by the necropsy results of goat no. 15, which died 9 dpv and whose organs were also analyzed by RT-qPCR. All organs were highly positive for PPRV genome and GEQ titers ranged between 10^3^/mL in the liver and 10^5^/mL in the other organs (data not shown). 

All samples of spleen and mediastinal and mesenterial lymph nodes from single rNDV_H_Kur_- vaccinated goats (group 4) were PPRV N positive, as well as one of the lung samples (range 10^2^–10^3^ GEQ/mL), whereas all liver samples were cleared. In contrast, none of the organ samples from double rNDV_H_Kur_-vaccinated goats (group 5) exhibited detectable amounts of PPRV N genome postmortem.

## 4. Discussion

The newly generated rNDV_H_Kur_, based on lentogenic NDV Clone 30 as a vector, expresses the surface attachment protein H of PPRV strain Kurdistan/11, which was confirmed by Western blot and confocal microscopic analyses of infected cells. Immunogenicity and protective efficacy of the recombinant ND vector virus was investigated after s.c. immunization of goats, either once or twice, in an interval of three weeks, followed by a severe PPRV challenge infection three weeks later. 

The pathogenicity of rNDV_H_Kur_ for one-day-old chickens was not altered compared to parental rNDV [62] by the insertion of PPRV H, an important prerequisite for NDV as a live vaccine. No sign of clinical disease was recorded in the goats after immunization. All twice vaccinated goats exhibited NDV NP specific antibodies as well as neutralizing antibodies against PPRV H three weeks after the second immunization, indicative of a certain level of replication of the avian virus at the site of inoculation into the goats (Figure 5A,C). This assumption was supported by in vitro replication kinetics, which demonstrated susceptibility of caprine cells to an NDV infection (Figure 3C). These observations match with results from immunizations of pigs, calves, lambs, or horses with recombinant NDV expressing either the glycoproteins of Nipah virus, Rift Valley fever virus, West Nile virus or bovine herpesvirus-1, respectively, and demonstrate the capacity of replication and immunogenicity of NDV in a mammalian host [54,55,56,57,76]. It was assumed that replication of NDV in mammals is locally restricted to the site of inoculation, which is supported by our findings since NDV RNA could not be found in the blood or nasal swabs after immunization [77]. 

Interestingly, goats, which were vaccinated twice with rNDV_H_Kur_, exhibited distinctly higher NDV seroconversion rates than control goats, which were likewise vaccinated twice, but with parental rNDV (Figure 5A). A similar effect was observed in calves, which were immunized with a recombinant NDV, expressing the glycoprotein Gn of Rift valley fever virus [56]. It was discussed that the foreign surface protein that was incorporated into the NDV envelope might be used as an additional receptor to infect cells, and consequently may induce a stronger immune response. 

However, no immune response was detected after a single s.c. immunization of goats with rNDV_H_Kur_, which might be caused by the route of virus administration (Figure 5A,C). Whereas in poultry the oculonasal application of NDV is preferred, since the respiratory epithelial cells represent the main target cells for NDV, it was shown that intranasal (i.n.) administration of an NDV vector is not sufficient to induce an immune response in calves [56]. The same observation was made with non-human primates [78]. Additionally, the delivery to the lower respiratory tract was discussed to be necessary. Whereas intramuscular (i.m.) or s.c. inoculation of pigs, calves, and sheep was shown to induce a proper immune response but requires a second application in an interval of three to four weeks, which matches with our observations, a combined i.n./intratracheal (i.t.) vaccination is discussed controversially. It was reported to be successful after a single administration in calves, but inferior to a parental application in sheep [53,54,59].

Corresponding to the serology, all twice-immunized goats were protected against a challenge infection with virulent PPRV Kurdistan/11 (Figure 6J). No hematogenic dissemination of the challenge virus was observed, as no PPRV RNA was found in the blood at any dpc or in the organs, which were investigated postmortem (Figure 7A and Figure 9). Accordingly, shedding via the nasal, ocular, oral, and fecal routes was significantly reduced, compared to goats that were either not vaccinated or double inoculated with parental rNDV (Figure 7B–E). The RT-qPCR data also indicate that the fever, exhibited by goat no. 06, was not related to the PPRV infection. 

In contrast, all non-immunized and rNDV-inoculated control goats developed PPR specific clinical signs including fever, as has been described for this isolate in German domestic goats before (Figure 6A,B,F,G) [70]. All control animals developed viremia, the challenge virus was shed in high amounts, detected by high viral loads in all swabs, and the animals were euthanized 9 or 14 dpc, as they had reached the humane endpoint (Figure 7). As expected, no significant differences were observed between non-vaccinated and rNDV-vaccinated animals, regarding the disease pattern. As *morbilliviruses* exhibit a pronounced lymphotropism [79], detection of PPRV RNA postmortem was most abundant in lymphatic tissue of the spleen and investigated lymph nodes. Additionally, the lungs as targets of primary PPRV infection were strongly positive and the liver could be identified as a site of viral replication (Figure 9). 

Although no immune response was detected after a single immunization with recombinant rNDV_H_Kur_, clinical disease was visibly reduced and one goat did not show any clinical signs at all, indicating that antibodies were present but not detectable by NDV-NP-ELISA or VNT (Figure 5A,C and Figure 6D,I). All goats of this group developed viremia and shed virus, but in a lower amount than non-vaccinated and rNDV-inoculated animals. However, all animals recovered and were healthy at the end of the trial (Figure 6D,I and Figure 7). In addition, viral shedding was reduced to a very low amount 28 dpc, while PPRV has been detected in the feces of non-vaccinated West African Dwarf goats for 12 weeks after a PPRV infection [80].

Goats vaccinated with the conventional live-attenuated PPRV vaccine Nigeria 75/1 were included in the trial to evaluate the results with the NDV recombinant. Two weeks after a single application of PPRV Nigeria 75/1, all goats seroconverted and were protected from a lethal PPRV Kurdistan/11 challenge infection (Figure 5B,C and Figure 6C,H). Since vaccination with PPRV Nigeria 75/1 induced neutralizing antibodies against the surface proteins F and H, the observed neutralizing antibody titers were higher three weeks after vaccination, compared to goats vaccinated with rNDV_H_Kur_, which developed neutralizing antibodies only against PPRV H. Whereas vaccination with PPRV Nigeria 75/1 was able to prevent viremia, viral RNA was detected in few amounts in some swabs taken after challenge infection, very similar to the results, obtained after a repeated vaccination with rNDV_H_Kur_ (Figure 7). These results confirm serological cross protection between the PPRV lineages II and IV as reported before [21], and indicate that the tested NDV recombinant is a suitable addition to the conventional live-attenuated PPRV vaccines. PPRV RNA that was detected in lymphatic tissues postmortem (spleen and lymph nodes) is assumed to be derived from the vaccine strain and not from the challenge strain and poses additional evidence for the pronounced lymphotropism of PPRV as RNA was still measurable after six weeks (Figure 9).

A main disadvantage of live-attenuated PPRV vaccines in developing countries is their low thermal tolerance. OIE recommends the storage and transport of the vaccine freeze-dried in Weybridge medium (2.5% (w/v) lactalbumin hydrolysate (LAH), 5% (w/v) sucrose, and 1% (w/v) sodium glutamate, in Hank’s balanced salt solution (HBSS)) at 2–8 °C [73]. The loss of infectious virus due to the process of lyophilization was shown to be 0.72 log_10_TCID_50_/mL for live-attenuated PPRV vaccine Sungri/96. Furthermore, storage of the lyophilisate at 25 °C reduces the half-live of viral activity to 1.83 days, to 10 h at 37 °C, and to 1.33 h at 45 °C. Stability can be increased by the use of different stabilizers [75,81]. In contrast, a reduction of the viral titer of rNDV_H_Kur_ by approx. 1000-fold was observed at 21 and 37 °C after 14 days of incubation without the use of any stabilizing media (Figure 4B). This indicates a much higher thermal tolerance of NDV, compared to PPRV that might be explained by the different body temperatures of the host species the viruses are adapted to. Whereas 39 °C is considered normothermic within goats, chickens exhibit a body temperature of 43 °C. Although a continuous cold chain is still advisable, NDV might have an advantage as a vector for vaccine viruses regarding their transport to PPR-endemic regions as our results indicate that rNDV_H_Kur_ is less sensitive to temperature variations than PPRV. In addition to the stability of a vaccine, the differentiation between vaccine virus and field virus is an important feature for vaccine virus development and sero-epidemiological surveillance. The immune response in goats after vaccination with PPRV Nigeria 75/1 and challenge infection with PPRV Kurdistan/11 is not distinguishable, as goats vaccinated with the live-attenuated PPRV develop antibodies against all viral proteins (Figure 5B,C and Figure 8A,B). In contrast, recombinant NDV_H_Kur_ expresses only the surface protein H of PPRV and therefore, vaccination can be serologically differentiated from wild type virus infection by using a PPRV H specific ELISA to verify vaccination coverage, and a PPRV N specific ELISA to detect wild type virus infection, which is the concept of a DIVA vaccine. The determination of PPRV-neutralizing antibodies in rNDV_H_Kur_-vaccinated animals confirmed vaccination while an immune response against PPRV N was lacking (Figure 5B,C). The PPRV immune response after challenge infection was stronger in single rNDV_H_Kur_-vaccinated goats than in goats vaccinated twice, reflecting the extent of protection in these groups (Figure 8A). A fast clearance and a minimal replication of the challenge virus causes a weaker immune response, as it was observed in goats vaccinated twice with rNDV_H_Kur_. Furthermore, a decline in antibody titers was observed within animals, vaccinated with rNDV_H_Kur_ while a steady level was observed within animals vaccinated with Nigeria 75/1 after challenge infection. This result may contrast the course of a primary immune response to the PPRV N antigen in animals, vaccinated with the recombinant NDV DIVA vaccine against the secondary immune response to the same antigen that proceeds in goats, vaccinated with the attenuated-live PPRV vaccine. This assumption is supported by the analysis of neutralizing antibodies, representing the PPRV H specific antibodies in goats, vaccinated with rNDV_H_Kur_. They increased rapidly after challenge infection and stayed at a stable high level until the end of the trial, 28 dpc (Figure 8B). In contrast, neutralizing antibodies in goats, vaccinated with PPRV Nigeria 75/1, were already detected with high titers after vaccination and were not boosted by the challenge infection. 

In addition to our recombinant NDV_H_Kur_, other recombinant viruses expressing either PPRV F and/or H were described, but only a few studies included a PPRV challenge infection. A recombinant fowlpox virus that expresses PPRV H or F was not able to elicit a significant immune response in goats after two inoculations [30]. In contrast, recombinant capripoxviruses (CPVs) expressing either PPRV H or F protected goats from a lethal PPRV challenge infection after a single application [26,27]. Unlike for NDV, goats might exhibit a pre-existing CPV immunity that interfered with the vaccine virus and required a booster vaccination to maintain protection from PPR [28,29]. Furthermore, vaccination of goats with a replication-defective human adenovirus or a recombinant vaccinia virus (MVA) that expresses PPRV H protected them from a lethal PPRV infection [30,35]. Two other vaccination studies demonstrated the induction of antibodies after a prime-boost vaccination i.m. or s.c. in goats with recombinant PPRV H expressing adenoviruses in an interval of three weeks. An oral and i.n. application was not able to induce an immune response [32,34]. In most of the cases, a prime-boost vaccination scheme was necessary for the induction of a significant immune response in the goats, which might also be dependent on the route of virus application. As discussed earlier, a combined i.n./i.t. application of NDV might have the potential to be even more potent after a single vaccine application.

## 5. Conclusions

In summary, we demonstrated in this proof-of-concept study that an NDV vectored PPR vaccine is safe and effective and can be developed into an alternative for s.c. vaccination in goats. The recombinant NDV_H_Kur_ protected goats from a lethal PPRV challenge infection. Furthermore, two vaccinations induced neutralizing antibodies and completely prevented clinical signs and viremia after challenge infection, reducing viral shedding via different routes. These results indicate that recombinant NDV vaccine viruses can assist in the process of eradication (e.g. in outbreak situations in formerly PPRV-free regions) as they provide DIVA-compatibility and a high thermal tolerance.

## Figures and Tables

**Table 1:** Experimental design of the animal trial.

Group		Goats	Vaccination			Challenge Infection	
			Vaccine Virus	Prime (Week)	Boost (Week)	Challenge Virus	Week
1	Negative control group	no. 25–29	-	-	-	PPRV Kurdistan/11	6
2	Empty vector control group	no. 20–24	rNDV	1	3		
3	Positive vaccine control group	no. 15–19 ^1^	PPRV Nigeria 75/1	3	-		
4	Test group	no. 10–14	rNDV_H_Kur_	3	-		
5	Test group	no. 05–09 ^2^	rNDV_H_Kur_	1	3		

^1^ Goat no. 15: Excluded from the trial on day 30. ^2^ Goat no. 09: Excluded from the trial on day 34.
